# Diabetic retinopathy and corneal endothelial parameters: an analytical cross-sectional study

**DOI:** 10.1186/s12886-022-02667-6

**Published:** 2022-11-09

**Authors:** Seyed-Ali-Akbar Mortazavi, Mohammadreza Akhlaghi, Alireza Dehghani, Mohsen Pourazizi, Mohammad Malekahmadi, Mohammadreza Fazel, Mehdi Tavakoli, Pegah Noorshargh

**Affiliations:** Isfahan Eye Research Center, Department of Ophthalmology, Isfahan University of Medical Sciences, Isfahan, Iran

**Keywords:** Diabetic retinopathy, Corneal endothelium, Diabetic mellitus, Specular microscopy

## Abstract

**Background:**

To investigate the possible association of different pattern of diabetic retinopathy (DR) on corneal endothelium cells in type 2 diabetes mellitus patients.

**Methods:**

In this descriptive-analytical cross-sectional study, corneal endothelium parameters including endothelial cell density (ECD), average cell size (AVG), coefficient of variation in cell size (CV), and hexagonality (Hex) were evaluated by non-contact specular microscopy.

**Results:**

One hundred and thirty-four eyes of 134 diabetic patients including 77 females (57.5%) with a mean age of 61.03 ± 8.08 years were enrolled. The overall corneal parameters in diabetic patients with and without retinopathy were not significantly different (*P* > 0.05). There is a significant relationship between CV and the duration of the disease with age variable control (B = 0.369, *p*-value < 0.001).

**Conclusions:**

Corneal endothelial parameters were not associated with DM in patients without and with DR. There is a significant relationship between CV and the duration of the disease with age variable control.

## Introduction

The morphological and functional integrity of the endothelial layer of the cornea is a critical factor for the maintenance of corneal clarity. Impairment in morphological or functional of the corneal endothelial layer is associated with increased risk of corneal decompensation due to susceptibility of the cornea to the recurrent corneal erosions, superficial keratitis, punctate epithelial keratopathy, persistent epithelial defects, recurrent ulceration following trauma or surgical insult [1–3].

Although diabetic retinopathy (DR) is one of the most important causes of blindness all over the world [4, 5], diabetes mellitus (DM) also affect the anterior segment element including corneal endothelium [6, 7]. DM can alter cell morphology, cell density, ultrastructure, barrier function, and finally the outcome of any intraocular surgery [8–10].

Currently, there is inconsistent evidence of whether DR and their severity may affect corneal endothelial indexes or not [11–13]. There are limited studies on the association of the severity of DR and corneal endothelium parameters [12]. This discrepancy may be related to type, severity, and duration of diabetes or type and severity of DR. The clinical importance of corneal endothelial indexes is related to important factors for the prediction of anterior segment surgery including cataract surgery outcomes and corneal transplant outcomes [8, 9, 14].

On one hand, DM causes structural and functional impairments of the corneal endothelium [9, 15], and on the other hand prevalence of anterior segment surgery including cataract surgery is high in these patients; so it seems pre-operative corneal assessment, in the diabetic population, is an important and rational evaluation.

This study aimed to investigate the association of the DR and their severity and related on the findings of specular microscopy in diabetic patients without and with different DR.

## Material and methods

### Study design and setting

This study is a descriptive-analytical cross-sectional study of the effect of DM and DR on corneal endothelial parameters. It was performed in the eye Feiz Hospital affiliated with the Isfahan University of Medical Sciences in Isfahan between April 2019 and June 2020. The study was conducted by the provisions of the Helsinki Declaration. This study was performed based on ethical code obtained from Isfahan University of Medical Sciences with the number IR.MUI.MED.REC.1399.709. Written consent was obtained from all participants in the study before enrolling in the study.

### Participants

Participants in the study included men and women with the ages of over 40 years who had a definite diagnosis of type 2 DM. Exclusion criteria included conditions affecting the health of corneal endothelium like the history of injection of intraocular anti-vascular endothelial growth factor medications in the last 3 months, history of intraocular surgery/laser, corneal dystrophy, history of ocular trauma, active or passive ocular inflammation, active or passive ocular infection, any history of glaucoma in the patient, pregnancy, and lactation.

### Ophthalmological examinations

Slit-lamp biomicroscopy was performed to evaluate the corneal and lens condition. Besides, a fundus exam by indirect ophthalmoscope was performed by an expert ophthalmologist. The patients were categorized into five subgroups based on the type of retinal involvement included: 1- diabetic patients without retinal involvement, 2- diabetic patients with mild and moderate non-proliferative DR (NPDR), 3- diabetic patients with severe NPDR, 4- diabetic patients with mild proliferative DR (PDR), 5- diabetic patients with high-risk PDR.

Early Treatment Diabetic Retinopathy Study (ETDRS) criteria were used as standardized guidelines for the interpretation of the various forms of DR [16].

In addition, patients were assessed by Non-contact specular microscopy (Tomey Corporation Inc, Nagoya, Japan) to evaluate the corneal endothelial cells.

Corneal endothelial cells parameters included endothelial cell density (ECD), average cell size (AVG), coefficient of variation in cell size (CV), and hexagonality (Hex). CV less than 40, Hex above 60, and cell density in the range of 1500–2500 were considered normal [8, 11].

### Statistical analysis

Data were analyzed using SPSS version 2020 (SPSS inc. Chicago IL). The results were expressed as mean ± standard deviation (SD) or as medians with ranges. Independent samples t-test was applied to compare the means of continuous variables. For continuous variables with skewed distributions, the Mann–Whitney *U* test was applied. Statistically significant differences were analyzed by the chi-square test for categorical variables. The differences among 3 or more groups were analyzed by one-way ANOVA. Also, the partial correlation coefficient that controlled for age was used to evaluate the correlation between the duration of the disease and corneal endothelial parameters. Patients were divided into different subgroups based on the existence and severity of DR and the condition of patients' corneal endothelial cells. A value of *P* ≤ 0.05 was considered statistically significant.

## Results

One hundred and thirty-four eyes of 134 diabetic patients, 40 without DR, and 94 with different degrees of DR were enrolled. The mean age of the patients was 61.03 ± 8.08 years and there were 77 females (57.5%). The median duration of diabetic disease was 10 [7,34] years. Table 1 presents patient demographics and clinical findings (Table 1).

**Table 1:** The demographic and clinical characteristics of the patients based on NPDR and PDR status

		**NPDR**		***P*-value**	**PDR**		***P*-value**
		**Mild to Moderate (*n* = 26)**	**Severe (*n* = 28)**		**Early (*n* = 21)**	**High risk (*n* = 19)**	
**Sex**	Female	14 (45.2)	17 (54.8)	0.61 ^Ω^	12(54.5)	10 (45.5)	0.75^Ω^
	Male	12 (52.2)	11(47.8)		9(50.0)	9(50.0)	
**Age** (years)	Mean ± SD	59.0 ± 5.58	60.39 ± 7.03	0.43^¶^	59.71 ± 5.51	63.21 ± 12.74	0.28^¶^
	Median [min,max]	59.5 [50,70]	60.0[45,75]		60[50,70]	61[49,95]	
**Duration of disease** (years)	Mean ± SD	8.72 ± 5.22	9.04 ± 4.41	0.81^¶^	10.38 ± 4.72	11.47 ± 5.35	0.59^**©**^
	Median [min,max]	8 [0,20]	8.5 [1,15]		10[4,20]	10 [5,20]	

**Ω** Resulted from chi square Test

©Resulted from Mann U Witny Test

^**¶**^ Resulted from Independent T Test

Comparison of the corneal parameters between the patients with and without DR are shown in Table 2 (Table 2). The overall corneal parameters in diabetic patients with and without retinopathy were not significantly different between the two groups (*P* > 0.05). In addition, age-wise stratification of the subjects had not shown a significant difference between the two groups (Table 2).

**Table 2:** Age-wise comparison of outcome measures in patients with and without diabetic retinopathy

	Age group (years)	Without Diabetic Retinopathy	With Diabetic Retinopathy	*p*-value
Endothelial Cell Density *(cell/mm2)*	≤ 55 (*n* = 34)	2461.22 ± 157.5	2437.92 ± 530.29	0.99^©^
		2501 [2190, 2665]	2431 [224, 3148]	
	55–60 (*n* = 19)	2483.5 ± 47.38	2612.71 ± 617.6	0.77^¶^
		2483.5 [2450, 2517]	2666 [531, 3564]	
	60–65 (*n* = 38)	2671.25 ± 311.61	2544 ± 265.8	0.20^¶^
		2527.5 [2325, 3354]	2543 [2001, 3037]	
	≥ 65 (*n* = 43)	2522.8 ± 317.92	2604.58 ± 281.20	0.38^¶^
		2460 [2138, 3353]	2639.5 [1944, 3015]	
	All age	2551.5 ± 284.41	2544.97 ± 428.20	0.33^©^
		2500.5 [2138, 3354]	2582.5 [224, 3564]	
Average cell size	≤ 55 (*n* = 34)	408.3 ± 27.17	401.08 ± 39	0.61^¶^
		400 [375, 457]	411 [318, 451]	
	55–60 (*n* = 19)	402.5 ± 7.78	459.12 ± 369.31	0.14^©^
		402.5 [397, 408]	375 [281, 404]	
	60–65 (*n* = 38)	378.3 ± 40.80	391.19 ± 48.40	0.43^¶^
		396 [298, 430]	389.5 [294, 500]	
	≥ 65 (*n* = 43)	398.94 ± 46.3	388.73 ± 47.03	0.49^¶^
		379 [332, 514]	379 [332, 514]	
	All age	395.05 ± 40.47	405.43 ± 160.46	0.31^©^
		399.5 [298, 468]	387 [281, 1883]	
Coefficient of variation	≤ 55 (*n* = 34)	40.89 ± 7.97	40.96 ± 7.66	0.98^¶^
		44 [27, 52]	40 [30, 60]	
	55–60 (*n* = 19)	45.5 ± 7.78	42.41 ± 6.31	0.53^¶^
		45.5 [40, 51]	44 [32, 51]	
	60–65 (*n* = 38)	40.5 ± 7.56	45.61 ± 8.17	0.07^¶^
		40 [32, 62]	44 [34, 65]	
	≥ 65 (*n* = 43)	46.53 ± 4.68	43.07 ± 7.11	0.08^¶^
		48 [35, 52]	42 [33, 68]	
	All age	43.4 ± 6.94	43.09 ± 7.52	0.82^¶^
		43.5 [27, 62]	43 [30, 68]	
Hexagonality (%)	≤ 55 (*n* = 34)	50.66 ± 9.06	46.8 ± 6.46	0.17^¶^
		50 [39, 65]	47 [33, 66]	
	55–60 (*n* = 19)	45.5 ± 6.36	45.12 ± 7.75	0.95^¶^
		45.5 [41, 50]	45 [30, 61]	
	60–65 (*n* = 38)	49.83 ± 9.98	46.46 ± 10.45	0.35^¶^
		45.5 [41, 77]	45.5 [29, 80]	
	≥ 65 (*n* = 43)	46.88 ± 11.48	46.19 ± 8.38	0.82^¶^
		48 [23, 67]	47.5 [29, 66]	
	All age	48.55 ± 10.16	46.23 ± 8.34	0.22^©^
		47.5 [23, 77]	46 [29, 80]	

© Resulted from Mann U Witny Test

^**¶**^ Resulted from Independent T Test

A comparison of the corneal parameters according to DR classification is shown in Table 3 (Table 3). In the 60–65 years’ age groups, statistically increased CV was seen with increasing the severity of DR. Mean CV were 40.5 ± 7.56, 43.56 ± 8.60, and 48.9 ± 6.54 in the patients without DR, NPDR, and PDR, respectively. The differences in CV between the groups were marginally significant (*P* = 0.052) (Table 3).

**Table 3:** Age-wise comparison of outcome measures in patients without DR, NPDR and PDR

	Age group (years)	Without DR (*n* = 40)	NPDR (*n* = 54)	PDR (*n* = 40)	*p*-value
Endothelial cell density *(cell/mm2)*	≤ 55 (*n* = 34)	2461.22 ± 157.5	2395.64 ± 685.96	2491.72 ± 239.81	0.87^¶^
		2501 [2190, 2665]	2431 [224, 3148]	2404 [2217, 2920]	
	55–60 (*n* = 19)	2483.5 ± 47.37	2512.5 ± 714.28	2853.2 ± 142.28	0.55^¶^
		2483.5 [2450, 2517]	2613 [531, 3564]	2834 [2665, 2998]	
	60–65 (*n* = 38)	2671.25 ± 311.62	2565.87 ± 277.8	2509 ± 255.85	0.39^¶^
		2527.5 [2325, 3354]	2543 [2168, 3037]	2563 [2001, 2892]	
	≥ 65 (*n* = 43)	2522.88 ± 317.93	2677.42 ± 212.40	2542.14 ± 323.72	0.35^¶^
		2460 [2138, 3353]	2693 [2164, 3015]	2585.5 [1944, 2944]	
	All age	2551.51 ± 284.4	2534.66 ± 512.47	2558.87 ± 282.80	0.95^¶^
		2500.5 [2138, 3354]	2582.5 [224, 3564]	2568.5 [1944, 2998]	
Average cell size	≤ 55 (*n* = 34)	408.33 ± 27.17	398.43 ± 41.88	404.45 ± 36.73	0.81^¶^
		400 [375, 457]	410 [318, 450]	416 [342, 451]	
	55–60 (*n* = 19)	402.5 ± 7.78	504.08 ± 436.78	351.2 ± 17.60	0.057^©^
		402.5 [397, 408]	382.5 [281, 404]	353.0 [334, 375]	
	60–65 (*n* = 38)	378.33 ± 40.79	386.63 ± 49.76	398.5 ± 47.76	0.60^¶^
		396 [298, 430]	387 [294, 461]	390 [320, 500]	
	≥ 65 (*n* = 43)	398.94 ± 46.30	375.75 ± 32.49	399.86 ± 55.42	0.33^¶^
		400 [298, 468]	371 [332, 462]	386.5 [340, 514]	
	All age	395.05 ± 40.47	413.37 ± 208.33	394.7 ± 47.02	0.43^©^
		399.5 [298, 468]	387 [281, 1883]	389 [320, 514]	
Coefficient of variation	≤ 55 (*n* = 34)	40.88 ± 7.97	40.71 ± 8.31	41.28 ± 7.05	0.98^¶^
		44 [27, 52]	38 [30, 60]	42 [31, 53]	
	55–60 (*n* = 19)	45.5 ± 7.78	43.17 ± 5.83	40.6 ± 7.73	0.63^¶^
		45.5 [40, 51]	44 [32, 51]	38 [32, 51]	
	60–65 (*n* = 38)	40.5 ± 7.56	43.56 ± 8.60	48.9 ± 6.54	0.052^¶^
		40 [32, 62]	43 [34, 65]	47.5 [40, 61]	
	≥ 65 (*n* = 43)	46.53 ± 4.67	43 ± 5.08	43.14 ± 8.68	0.23^¶^
		48 [35, 52]	43 [34, 51]	41.5 [33, 68]	
	All age	43.4 ± 6.94	42.6 ± 7.519	43.7 ± 7.98	0.74^¶^
		43.5 [27, 62]	43 [30, 65]	43.5 [31, 68]	
Hexagonality (%)	≤ 55 (*n* = 34)	50.66 ± 9.06	46.78 ± 5.52	49.27 ± 13.72	0.41^¶^
		50 [39, 65]	47.5 [30, 59]	49 [32, 80]	
	55–60 (*n* = 19)	45.50 ± 6.36	44.16 ± 6.64	47.4 ± 10.45	0.74^¶^
		45.5 [41, 50]	44 [30, 57]	49 [32, 61]	
	60–65 (*n* = 38)	49.83 ± 9.98	47.25 ± 6.96	45.2 ± 14.83	0.58^¶^
		45.5 [41, 77]	46 [35, 59]	40 [29, 80]	
	≥ 65 (*n* = 43)	46.88 ± 11.48	46.75 ± 6.01	45.71 ± 10.19	0.94^¶^
		48 [23, 67]	46.5 [37, 61]	49 [29, 66]	
	All age	48.55 ± 10.16	46.33 ± 6.26	46.1 ± 10.62	0.74^©^
		47.5 [23, 77]	46 [30, 61]	46.5 [29, 80]	

© Resulted from Kruskal–Wallis

^**¶**^ Resulted from One-Way ANOVA

Using a partial correlation coefficient, the relationship between corneal endothelial parameters and the duration of diabetes disease was investigated. There is a significant relationship between CV and the duration of the disease with age variable control (correlation = 0.326, *p*-value < 0.001) (Table 4).

**Table 4:** Partial correlation coefficients controlling for age in DR group

Adjusted Age	Endothelial cell density	Cell size	Coefficient Of variation	Hexagonality
Duration disease	0.025	0.140	0.369	0.041
*P*- value	0.81	0.18	< 0.001	0.69

Logistic regression analysis showed that there was no significant association between endothelial parameters and DR (Table 5).

**Table 5:** Logistic regression of related factors in patients with DR

Characteristic	B	SE	OR*	95% CI**	*P*-Value
Gender, (Male)	0.138	0.395	1.148	0.529–2.489	0.73
Age	-0.031	0.024	0.969	0.925–1.015	0.19
Endothelial cell density	0.00	0.001	1.00	0.999–1.002	0.74
Average cell size	0.001	0.003	1.00	0.996–1.007	0.66
Coefficient of variation	-0.011	0.027	0.989	0.938–1.043	0.69
Hexagonality	-0.031	0.022	0.970	0.928–1.013	0.16

OR* odds ratio

CI** confidence interval

## Discussion

The result of our study demonstrated that in diabetic patients without and with DR, corneal endothelial parameters were not statistically significant difference. There was a significant relationship between CV and the duration of the disease with age variable control.

The cornea with altered morphology and functionality is known to be more susceptible to pathologies like recurrent corneal erosions, and impaired corneal sensitivity following trauma or surgical insult leading to recurrent ulceration with impaired healing [1, 2, 17]. So recognition of any potential endothelial dysfunction before the surgery potentially can be associated with more positive surgical outcomes [18]. Corneal endothelial cell parameters can be helpful indexes before referring patients for cataract or refractive surgery [9, 18].

The possible explanation for corneal endothelial changes in DM patients is multifactorial including impairment of apical junctions on the endothelial cells, impairment of physical barriers of corneal cell and altered permeability of corneal cell due to reduced Na + /K + ATPase activity pump in the endothelial cells [19, 20] Diabetic cornea especially with high glucose can lead cellular swelling due to increased sorbitol inside the cells due to increased activity of aldose reductase [19, 21].

Having exact data about the number and morphology of endothelial cells before cataract surgery reduces the risk of endothelial injury, especially in patients with DM [11]. In contrast to our study, limited studies have addressed the association of the severity of DR with the altered corneal endothelial parameters [12, 13].

A recent study by Ashok Jha et al. demonstrated DM patients had significantly an altered morphology including increased polymegathism, decreased cell density, and hexagonality when compared with healthy controls [12]. The effect of severity of DR and corneal parameters can be indirect via the effect of factors like duration, the severity of DM, age, etc. [12].

Since there was no endothelial cells proliferation with aging, on one hand, the number of corneal endothelial will be decreased and on the other hand cells size will be increased to compensate for the lack of lost cells.

In each ocular surgery, the CV number should be in the normal range to ensure that it does not occur decompensation after the ocular surgery [8, 9]. In the current study, it was shown that there were no significant differences in CV number in NPDR and PDR groups.

Choo et al. in 2010, did not show any correlation to the duration of DM, hemoglobin A1c level, and severity of DR [12]. In contrast to the study of Choo et al., in our study, there is a significant relationship between CV and the duration of the disease with age variable control that is a predictable and acceptable finding regarding increasing the adverse effect of DM in all organs with increasing the duration of diabetes [22]. This differentiation may be due to different ethnicity between Iranian and Japanese populations and differences in duration of disease in the enrolled population.

The findings of a study of Nurdan Gamze Taşlı et.al [23] about corneal specular microscopy in patients with type-2 DM demonstrated an increase in the stage of DR, alterations in corneal findings also increased. In our study, marginally association was obtained in the 60–65 years age groups for CV.

The possible explanation for the absence of statistically significant differences between other parameters of endothelial changes and severity of DM can be attributed to a relatively small sample size of our study and may be associated with ethnic differences [23].

Although existence of DR is important factor for alternation of corneal endothelial parameters, factor associated with clinical course of diseases are important factors for this alternation. The finding of Yoo Jin Kim and Tae Gi Kim suggest that DM affects corneal endothelial cell in older age and those with long-standing DM and higher HbA1c [24].

The importance of our study lies on evaluation of possible association of diabetic retinopathy with corneal endothelial parameters in diabetic patients. In most previous study with case–control design, normal population considered as a control group but in our study in both group the patient had DM and existence of retinopathy was as independent variable.

The study of El-Agamy et al. included patients without DR, eyes with NPDR, and PDR. The results of their study demonstrated ECD was significantly lower in the diabetic cornea than in control group and CV was higher in diabetic cornea. The diabetic cornea group had lower percentage of hexagonal cells than the control group, but the difference was not statistically significant [25].

Although our study has a suitable data analysis regarding different age groups and different DR grades for evaluation of DR on corneal endothelial parameters effect, there is some limitation including relatively small sample size, absence of normal population group as normal control, absence of level of glycosylated hemoglobin (HbA1c) and absence of data about corneal thickness.

## Conclusion

The results of the current study demonstrated that DM has negative effects on a CV as one of the important corneal endothelium parameters. There is a significant relationship between CV and the duration of the disease with age variable control. So, the long-lasting DM may further warrant a corneal evaluation before intraocular surgery.

## Data Availability

All data generated or analyzed during this study are included in this published article.

## Abbreviations

DR: Diabetic retinopathy
ECD: Endothelial cell density
AVG: Average cell size
CV: Coefficient of variation in cell size
Hex: Hexagonality
DM: Diabetes mellitus
ETDRS: Early treatment diabetic retinopathy study
SD: Standard deviation
